# CUL4 E3 ligase regulates the proliferation and apoptosis of lung squamous cell carcinoma and small cell lung carcinoma

**DOI:** 10.20892/j.issn.2095-3941.2019.0107

**Published:** 2020-05-15

**Authors:** Ting Li, Si Wu, Lei Jia, Wenfeng Cao, Yuan Yao, Gang Zhao, Hui Li

**Affiliations:** ^1^Department of Gastrointestinal Cancer Biology, Tianjin Medical University Cancer Institute and Hospital, National Clinical Research Center for Cancer, Key Laboratory of Cancer Prevention and Therapy, Tianjin, Tianjin’s Clinical Research Center for Cancer, Tianjin 300060, China; ^2^Key Laboratory of Cancer Immunology and Biotherapy, Tianjin Medical University Cancer Institute and Hospital, National Clinical Research Center for Cancer, Key Laboratory of Cancer Prevention and Therapy, Tianjin, Tianjin’s Clinical Research Center for Cancer, Tianjin 300060, China; ^3^Department of Pathology, Tianjin Medical University Cancer Institute and Hospital, National Clinical Research Center for Cancer, Key Laboratory of Cancer Prevention and Therapy, Tianjin, Tianjin’s Clinical Research Center for Cancer, Tianjin 300060, China

**Keywords:** Squamous cell lung cancer, small cell lung cancer, CUL4A, CUL4B, P21, FOXO3A

## Abstract

**Objective:** The E3 ligase, CRL4, plays diverse roles in different cellular processes, such as DNA damage, transcriptional regulation, cell cycle progression, and cell apoptosis. Our previous study showed that CUL4A and CUL4B had a strong association with tobacco smoking risk in lung squamous cell carcinoma (SCC) and small cell lung carcinoma (SCLC). This study aimed to define the potential mechanism underlying the roles of CUL4A and CUL4B in the development of SCC and SCLC.

**Methods:** We determined the role of CUL4A and CUL4B in the cell cycle and apoptosis of SCC and SCLC, and identified the key apoptosis-related gene involved in the oncogenic activity of CUL4B by Western blot, immunohistochemical staining, flow cytometry, and enzyme inhibition experiments.

**Results:** We found that depletion of CUL4A and CUL4B reduced the proliferation of SCC and SCLC cells. CUL4A^knockdown^ but not CUL4B^knockdown^ arrested cells in G1 phase while upregulating P21 and CUL4B^knockdown^ promoted cell apoptosis through upregulation of FOXO3A. Accordingly, CUL4B decreased FOXO3A expression by activating the ERK signaling pathway and mediating FOXO3A degradation *via* the ubiquitin-proteasome pathway.

**Conclusions:** These results identified the function of E3 ligase CRL4 in regulating SCC and SCLC cell proliferation, which provides a potential strategy for cancer therapy by targeting FOXO3A and the E3 ligase, CRL4.

## Introduction

Ubiquitination is a posttranslational modification, which is involved in modulating the activity of most eukaryotic proteins and many biological processes. The roles of ubiquitination in cancer-related pathways have been identified in recent years, including its involvement in cell cycle progression, cell apoptosis, and gene transcriptional regulation^1–3^. First, ubiquitination is reported as a process used to mark target proteins for degradation in the proteasome, and all members of the process form the ubiquitin-proteasome system (UPS). Recently, ubiquitination involvement has been shown to span the processes of modulating proteins to regulating the activities of proteins, which leads to other biological effects, such as DNA damage and the immune response^4–6^.

E3 ubiquitin ligases are considered to be central to the ubiquitin-conjugation system because they directly and specifically interact with substrates, acting as scaffold proteins that interact with the E2 enzyme and transfer ubiquitin to the target protein^7^. E3 ligases have been classified into two structurally and functionally distinct families: the HECT ligases and the RING/Ubox ligases^8^. Among the RING/Ubox ligases, the cullin-RING ubiquitin ligases (CRLs) constitute the largest E3 ligase family in eukaryotes^9^. Eight proteins comprise the human cullin family, including CUL1, CUL2, CUL3, CUL4A, CUL4B, CUL5, CUL7, and CUL9. The CUL4 subfamily has two members, CUL4A and CUL4B, which share over 80% sequence identity and functional redundancy. Specific adaptors and diverse substrate receptors serve different cullins. In particular, DNA damage binding protein 1 (DDB1) is an adaptor and CUL4-associated factors (DCAFs) are substrate receptors, and both have been used to identify a large number of CUL4 substrates^9–11^. Substrates of CUL4 play diverse roles in tumor progression, including DNA repair, cell cycle arrest, cell apoptosis, cell invasion, and cell migration^12–15^. The aberrant expression of CUL4 has been reported in breast cancer and hepatocellular cancer^16,17^. CUL4B has been negatively correlated with IGFBP3 expression, which enhances the p53-dependent apoptotic response^18^.

Our previous study showed that high expression of CUL4A and CUL4B was significantly associated with worse clinicopathological characteristics, including lymph node metastasis, higher tumor node metastasis stage, distant metastasis, and a larger tumor size in different subtypes of lung cancer, including squamous cell carcinoma (SCC), adenocarcinoma, large cell carcinoma, and small cell lung carcinoma (SCLC). The expression of P21 and XPC is negatively associated with CUL4A in lung cancer. In particular, we observed a strong association of high CUL4 levels with tobacco smoking risk in SCLC and SCC, the two smoking-related lung cancer subtypes^19^. Therefore, we characterized the effects and underlying mechanisms of CUL4A and CUL4B in SCLC and SCC in this study.

CUL4A limits DNA repair through ubiquitin-dependent proteolysis of the rate-limiting NER factors, DDB2 and XPC. The cyclin-dependent kinase inhibitor, P21, which delays S phase entry by inhibiting the cyclin E/Cdk2 complex, is a known substrate of CUL4A^20^. The effects of CUL4A on the cell cycle through P21 in SCLC and SCC were verified in this study. In addition, CUL4B was reported to coordinate with PRC2 in H3K27me3-mediated transcriptional repression. FOXO3A was transcriptionally repressed by PRC2^21^. Phosphorylation by Akt, IkB kinase, and ERK also reduces FOXO protein abundance through FOXO polyubiquitination and proteasome-dependent degradation^22–24^. FOXO3A can transcriptionally promote apoptosis-correlated protein expression, such as the expression of Bim^25,26^. Whether CUL4B exerts an important role in cell apoptosis through ubiquitination and degradation of FOXO3A was determined in the current study. Finally, we found that CUL4A downregulated P21 to promote cell cycle progression, and that CUL4B facilitated cancer cell survival by downregulating FOXO3A by accelerating the degradation of p-FOXO3A.

## Materials and methods

### Cell lines, antibodies and reagents

Human SCC cell lines, NCI-H520 and SK-MES-1, and human SCLC cell lines, DMS114 and NCI-H2227, were obtained from the American Type Culture Collection (Manassas, VA, USA). Antibodies were purchased from commercial sources, including rabbit anti-CUL4B antibody (1:50; Novus Biologicals, Littleton, CO, USA), rabbit anti-CUL4A (1:1,000; Cell Signaling Technology, Danvers, MA, USA), rabbit anti-FOXO3A (1:1,000; Cell Signaling Technology), rabbit anti-p-FOXO3A (1:1,000; Cell Signaling Technology), rabbit anti-cyclin E1 (1:1,000; Abcam, Cambridge, UK), rabbit anti-ERK (1:1,000; Abcam), rabbit anti-p-ERK (1:1,000; Abcam), rabbit anti-P21 (1:1,000; Proteintech, Rosemont, IL, USA), rabbit anti-CUL4B (1:1,000; Proteintech), rabbit anti-BCL-2 (1:1,000; Proteintech), rabbit anti-BAX (1:1,000; Proteintech), rabbit anti-caspase 3 (1:1,000; Proteintech), rabbit anti-UBC12 (1:1,000; Proteintech), and mouse anti-β-actin (1:1,000; Sigma-Aldrich, St. Louis, MO, USA). Si-RNAs were purchased from RiboBio (Guangzhou, China), including si-FOXO3A and si-UBC12. MG132, MLN4924, and PD98059 were purchased from Selleck Chemicals (Houston, TX, USA). Cycloheximide (CHX) was purchased from Absin (Shanghai, China).

### Plasmid construction, transfection and lentiviral infection

ShRNA plasmids for knocking down CUL4A and CUL4B were described previously^27^. To generate the recombinant sh-CUL4A, sh-CUL4B, or sh-nontarget (sh-NT), lentiviruses were used. The 293T cells in 10 cm dishes were transfected with 12 μg of shRNA, 9 μg of Δ8.9, and 6 μg of VSV-G plasmids using the Lipofectamine® 2000 transfection reagent (Invitrogen, Carlsbad, CA, USA). After 48 h of transfection, the supernatants were collected for infection of SCC and SCLC cells. Infected NCI-H520 and SK-MES-1 cells were selected in RPMI-1640 medium (Gibco, Thermo Fisher Scientific, Waltham, MA, USA), NCI-H2227 in Dulbecco’s Modified Eagle Medium:F12 (Gibco, Thermo Fisher Scientific), and DMS114 in Waymouth Medium (Gibco) containing 4 μg/mL puromycin dihydrochloride (Sigma-Aldrich, Buchs, Switzerland).

### Cell proliferation, cell cycle arrest and cell apoptosis assays

The number of cells in the culture was used to evaluate the growth ability of cells. The cells were cultured in 24-well plates at a density of 1 × 10^4^ cells/well (NCI-H520 and NCI-H2227) or 2 × 10^4^ cells/well (SK-MES-1 and DMS114). Cell counts were obtained from all groups at 24, 48, 72, and 120 h involving three repetitions, with the wells counted three times. For the cell cycle, three repetitions were measured for each group. The cells were harvested and fixed overnight using ethanol. The cells were then washed once with phosphate-buffered saline (PBS), diluted in 100 μL PBS, and stained using propidium iodide (PI) at 4 °C for 30 min. For apoptosis, there were also three repetitions in each group. The cells were washed with PBS and then resuspended in 100 μL annexin V-binding buffer, followed by staining with FITC-conjugated annexin V and PI at 4 °C for 30 min. Finally, 200 μL PBS or annexin V-binding buffer was added to each sample to make a final volume of 300 μL before analysis by flow cytometry.

### Western blot

Total cell lysates were obtained using RIPA buffer, and extracted using centrifugation at 18,000 × *g* at 4 °C for 25 min. The proteins for Western blot were denatured by 5× Tris-acetate sample buffer at 95 °C for 5 min. The protein concentration was determined using a bicinchoninic acid protein assay kit (Sigma-Aldrich). The samples (20 μg) were separated by 10% SDS-PAGE and transferred to polyvinylidene difluoride membranes (Bio-Rad, Hercules, CA, USA). Following blocking with 5% nonfat milk for 1 h in Tris-buffered saline with 0.05% Tween-20 buffer (TBST) at room temperature, the membranes were incubated overnight with primary antibody at 4 °C. Following TBST washing, the membranes were incubated for 2 h with horseradish peroxidase-conjugated goat anti-rabbit secondary antibody (Thermo Fisher Scientific). The protein bands were detected and visualized using an electrochemiluminescence system (Amersham Imager 600; GE Healthcare Bio-Sciences, Piscataway, NJ, USA).

### The cycloheximide (CHX), MG132 chase, and MEK inhibitor experiments

Cells were infected with sh-CUL4B or sh-NT lentiviruses, treated with 2 mM CHX, 10 μM MG132, and 20 μM PD98059, and then subjected to Western blot analysis.

### Immunohistochemical staining, scoring analysis and human subjects approval

This study was approved by the ethics committee of Tianjin Medical University Cancer Institute and Hospital. All patients in this study (or their legal representative) signed an informed consent document.

Serial sections (4 μm) were processed for immunohistochemical staining as follows. The sections were heated in a microwave oven at 65 °C for 2 h, deparaffinized by xylene, and dehydrated by a gradient of ethanol. After washing, the sections were incubated in sodium citrate buffer (pH = 6.0) for 3 min in an autoclave at 120 °C, then cooled to room temperature (RT) for antigen retrieval for at least 30 min. Endogenous peroxidase activity was blocked with 3% hydrogen peroxide for 20 min at RT. After washing with PBS, 3 times, nonspecific binding sites were blocked with normal goat serum for 10 min at RT. The sections were then incubated overnight at 4 °C with primary antibody. After washing with PBS, 3 times, the sections were incubated with secondary antibodies (Zhongshan Goldenbridge Biological Technology, Beijing, China) for 60 min at 37 °C. The sections were then washed 3 times with PBS and visualized with diaminobenzidine tetrahydrochloride (DAB kit; Zhongshan Goldenbridge Biological Technology, Beijing, China). Finally, the sections were counterstained with hematoxylin and dehydrated.

A two-way scoring system was used for staining quantification^28^. The staining intensity was scored in 4 categories: negative, 0; weak, 1; moderate, 2; and strong, 3. The percentages of positively stained cells of interest were determined as follows: 0–25%, 1; 26%–50%, 2; 51%–75%, 3; and 76%–100%, 4. The final expression levels of the proteins of interest in each sample were obtained by multiplying the proportion and the intensity for each protein.

### Statistical analysis

Statistical analysis was performed using GraphPad Prism software (GraphPad, San Diego, CA, USA). Comparisons between groups were analyzed using two-tailed Student’s *t*-test or one-way analysis of variance, as appropriate. Data were considered significant at the level of *P* < 0.05. Levels of significance are represented with asterisks as follows: *P < 0.05; **P < 0.01; and ***P < 0.001. The correlations between variables were determined by Spearman’s nonparametric correlation analysis.

## Results

### Depletion of CUL4A and CUL4B compromises cell proliferation

**Figure 1:**
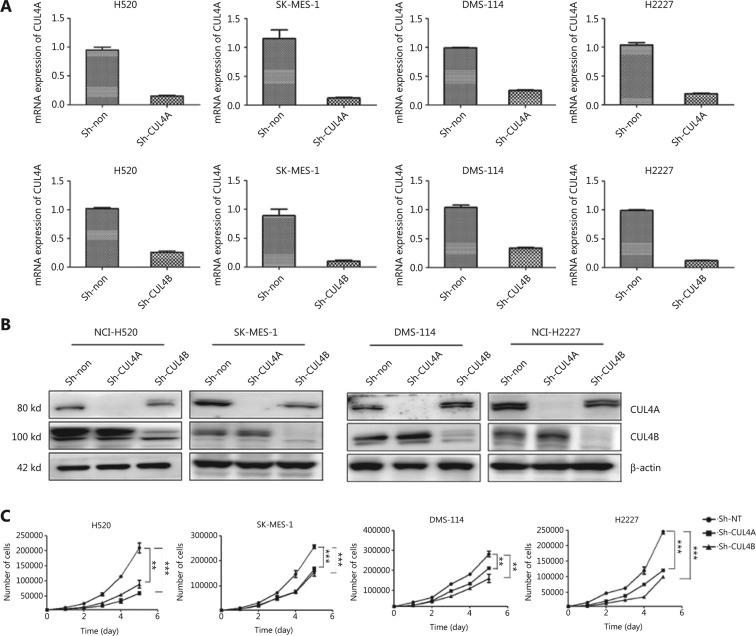
Depletion of CUL4A or CUL4B in squamous cell carcinoma (SCC) and small cell lung carcinoma (SCLC) cell lines compromises cell proliferation. To validate the depletion of CUL4A and CUL4B, qPCR was used to detect the mRNA levels of CUL4A or CUL4B (A), and the protein levels of CUL4A or CUL4B (B) were also detected by Western blot. We compared the growth curves of cells in the Sh-NT, Sh-CUL4A, and Sh-CUL4B groups (C). When levels of CUL4A or CUL4B were depressed, the cell growth rates were significantly decreased (^**^P < 0.01; ^***^P < 0.001).

To investigate the function of CUL4A and CUL4B in SCC and SCLC cells, the expression of CUL4A or CUL4B was knocked down by shRNAs specific to CUL4A and CUL4B. The results showed that both CUL4A and CUL4B mRNAs and proteins were reduced (**Figure 1A and 1B**). Stably expressing CUL4A or CUL4B knockdown cell lines were generated, and sh-NT cells were used as a control. To determine whether knockdown of either CUL4A or CUL4B affected cell proliferation, we counted the number of cells in 24-well plates to assess cell proliferation at 24, 48, 72, 96, and 120 h after seeding the cells. The results showed that cell proliferation was reduced, and the difference remained statistically significant at 120 h (**Figure 1C**). These results suggested that the expression of CUL4A and CUL4B promoted SCC and SCLC cell proliferation.

### The effects of CUL4A and CUL4B on cell cycle and apoptosis in SCC and SCLC cells

**Figure 2:**
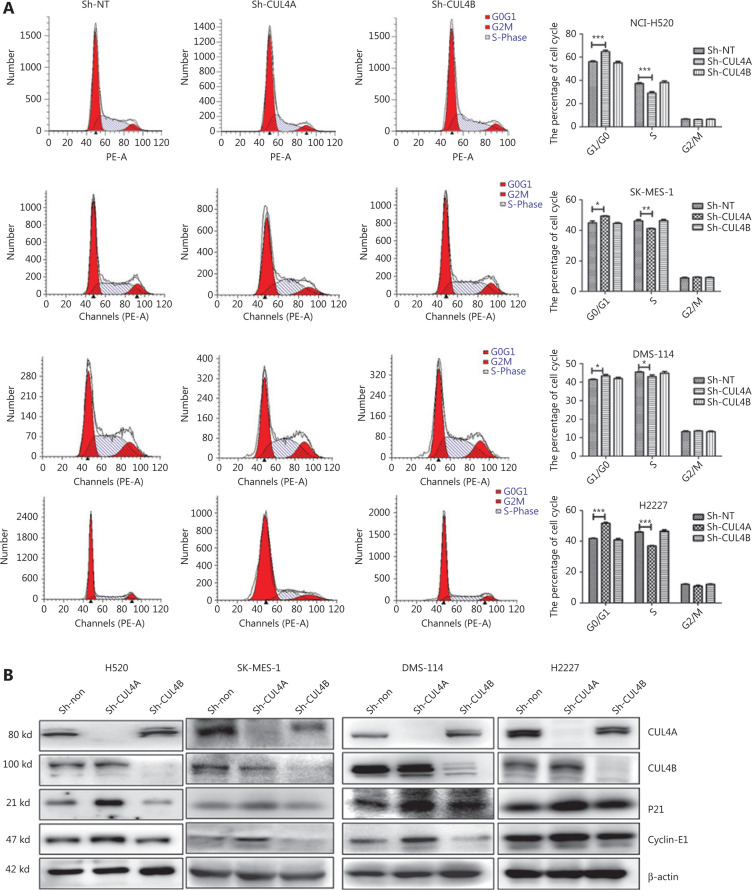
Different effects of CUL4A^knockdown^ and CUL4B^knockdown^ cells on cell cycle regulation. Propidium iodide staining showed that CUL4A^knockdown^, but not CUL4B^knockdown^ cells, arrested the cell cycle in G1 phase (left panel in A). Statistical analysis of the cell cycle is displayed in the right panel in A (^*^P < 0.05; ^**^P < 0.01; ^***^P < 0.001). Correspondingly, CUL4A^knockdown^ resulted in an increase of P21 and cyclin-E1 (B).

**Figure 3:**
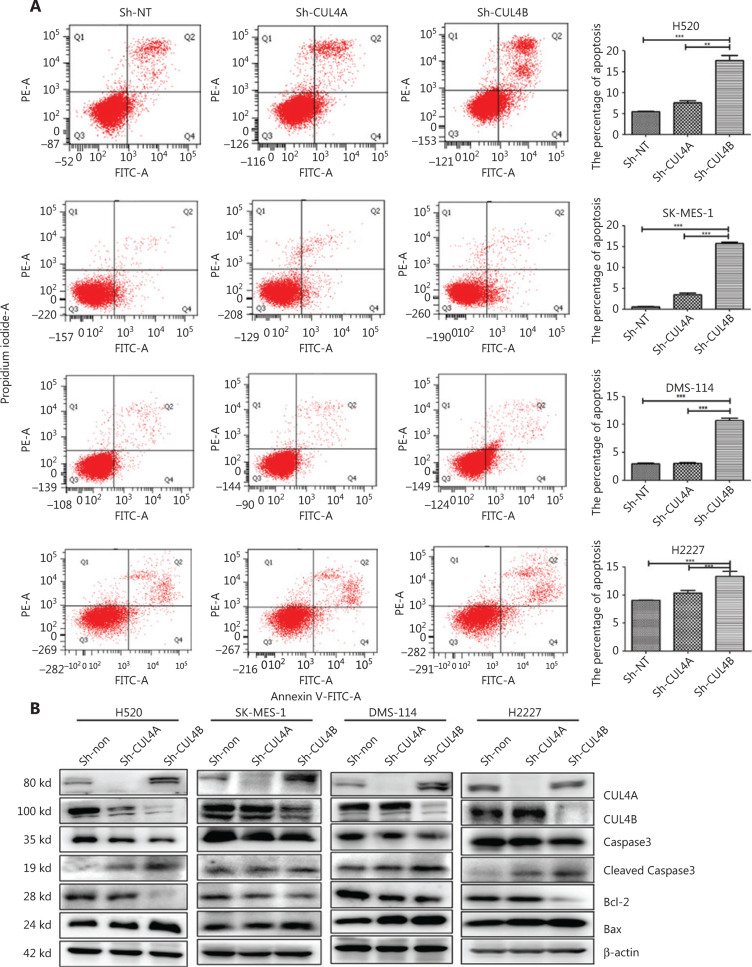
Different effects of CUL4A^knockdown^ and CUL4B^knockdown^ on cell apoptosis regulation. Annexin V/propidium iodide double staining showed that CUL4B^knockdown^, but not UL4A^knockdown^ cells, promoted cell apoptosis (left panel in A). Statistical analysis of apoptosis displays are shown in the right panel in A (^*^P < 0.05; ^**^P < 0.01; ^***^P < 0.001). Correspondingly, CUL4B^knockdown^ resulted in an increase of cleaved caspase-3 and BAX, and a decrease of BCL2 (B).

To further elucidate the mechanism of growth inhibition by CUL4A/CUL4B downregulation, flow cytometry was used to analyze the cell cycle and cell apoptosis in SCC and SCLC cells. Analysis of the cell cycle distribution showed that compared with the sh-NT and sh-CUL4B groups, CUL4A^knockdown^ significantly increased the number of cells in the G1 phase and decreased the number of cells in the S phase, whereas CUL4B^knockdown^ did not (**Figure 2A**). Moreover, the apoptotic percentage data showed that depletion of CUL4B led to a significant increase in the apoptotic percentages of SCC and SCLC cells, whereas depletion of CUL4A did not (**Figure 3A**). In addition, the apoptosis-associated proteins (BCL2, BAX, caspase-3, and cleaved caspase-3) were detected by Western blot. The results showed that the expression of apoptosis proteins (BAX and cleaved caspase-3) was increased, but expression of the anti-apoptosis protein (BCL-2) was reduced by CUL4B^knockdown^ (**Figure 3B**). Taken together, these data suggested that downregulation of CUL4A and CUL4B inhibited SCC and SCLC cell proliferation by inducing G1-phase cell cycle arrest and promoting apoptosis, respectively.

### CUL4A^knockdown^ induces G1 phase arrest by P21 in lung cancer cells

**Figure 4:**
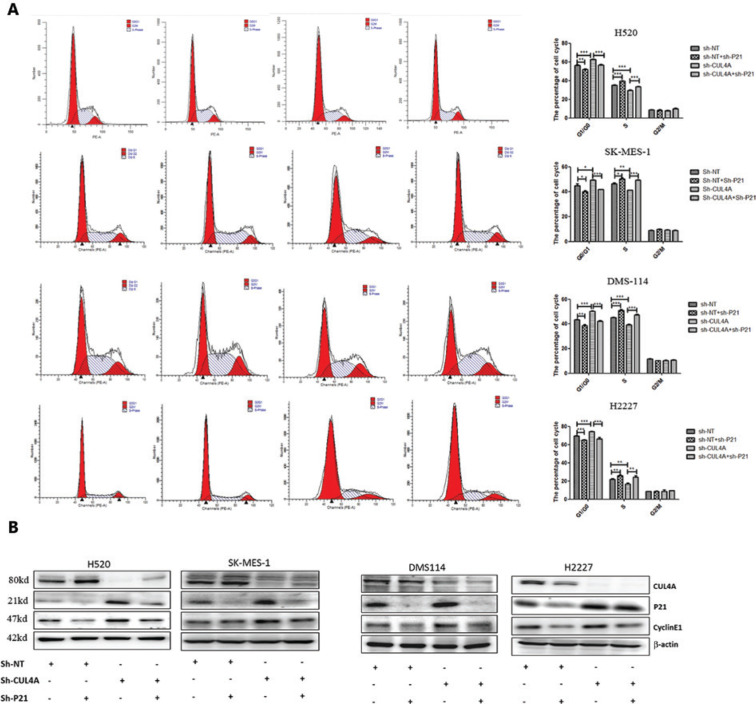
Depletion of P21 in CUL4A^knockdown^ cells releases cell cycle arrest. Propidium iodide staining showed that the cell cycle arrest in CUL4A^knockdown^ cells was released by depletion of P21 (left panel in A, from left to right: Sh-NT group, Sh-NT+Sh-P21 group, Sh-CUL4B group, and Sh-CUL4B+Sh-P21 group). Statistical analysis of cell cycle is displayed in the right panel in A (^*^P < 0.05; ^**^P < 0.01; ^***^P < 0.001). The increase of P21 and cyclin-E1 in CUL4A^knockdown^ cells was partially reversed by the depletion of P21 (B).

P21 is a substrate of CUL4A^20^, which arrests the cell cycle and ensures genomic stability during various cellular stress reactions by inhibiting the activation of the CDK/cyclin complex. To assess whether P21 played a role in CUL4A^knockdown^-induced G1 phase arrest, P21 and cyclin E1 expression was detected at the protein level by Western blot. As shown in **Figure 2B**, P21 and cyclin E1 were increased after depletion of CUL4A. To confirm the function of P21 and CUL4A in cell cycle arrest, we further knocked down P21 in the stably expressed SCC and SCLC cells with sh-NT and sh-CUL4A. With the depletion of both CUL4A and P21, G1 phase arrest was attenuated, and cyclin E1 expression was recovered (**Figure 4**). These results suggested that CUL4A suppression modulated the SCC and SCLC cell cycle *via* upregulation of P21.

### CUL4B^knockdown^ enhances apoptosis by FOXO3A

**Figure 5:**
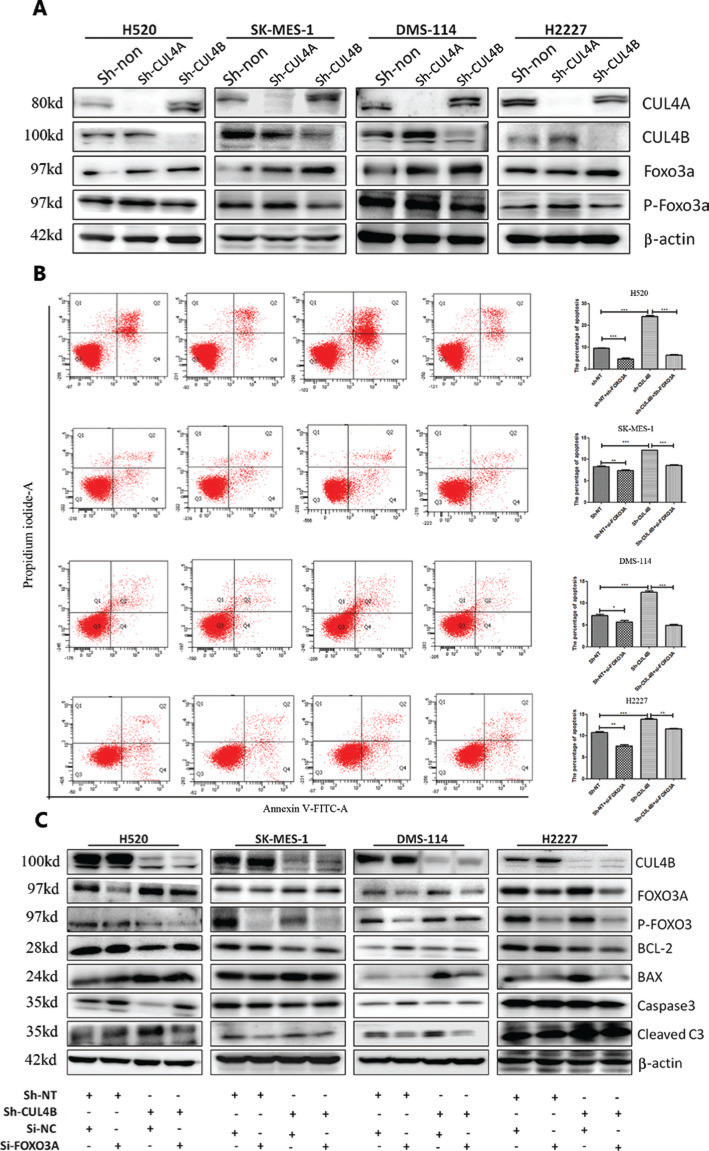
The increase of FOXO3A promotes cell apoptosis in CUL4B^knockdown^ cells. FOXO3A was upregulated by the depletion of CUL4B (A). The increase of cell apoptosis in CUL4B^knockdown^ cells was recovered by the knockdown of FOXO3A (B) (left panel in B, from left to right: Sh-NT+si-NC group, Sh-NT+si-FOXO3A group, Sh-CUL4B+si-NC group, and Sh-CUL4B+si-FOXO3A group). Statistical analysis of cell apoptosis is displayed in the right panel in B (^*^P < 0.05; ^**^P < 0.01; ^***^P < 0.001). The knockdown of FOXO3A resulted in decreases of cleaved caspase-3 and BAX, and an increase of BCL2 (C).

Apoptosis can be transcriptionally promoted by FOXO3A, which is suppressed by CUL4B in H3K27me3-mediated transcriptional repression. To determine whether FOXO3A is involved in cell apoptosis regulation, we determined FOXO3A protein expression in SCC and SCLC cells with stable knockdown of CUL4B. The results showed that FOXO3A was increased with cleaved caspase-3 and BAX upregulation in CUL4B^knockdown^ cells (**Figure 5A**). To further determine whether the upregulation of FOXO3A was a necessary factor in CUL4B^knockdown^-enhanced apoptosis, we transfected siRNA against FOXO3A into SCC and SCLC cells with stable knockdown of CUL4B, and determined whether apoptosis could be rescued. FOXO3A knockdown in CUL4B-depleted cells resulted in a significant decrease in the percentage of apoptotic cells (**Figure 5B**). The expression levels of cleaved caspase-3, BAX, and BCL-2 were recovered after FOXO3A knockdown (**Figure 5C**). These results showed that CUL4B mediated SCC and SCLC cell apoptosis through FOXO3A.

### The mechanism by which CUL4B depletion induces FOXO3A upregulation

**Figure 6:**
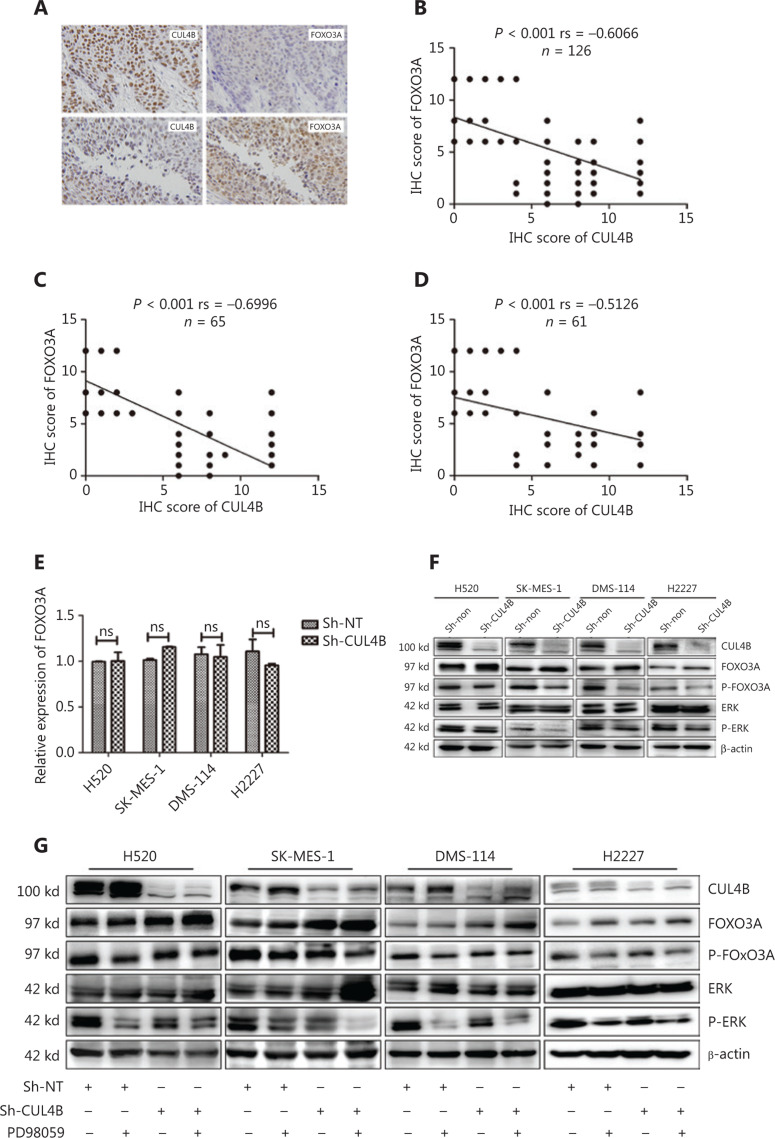
The relationship between CUL4B and FOXO3A in squamous cell carcinoma (SCC) and small cell lung carcinoma (SCLC) tissues, and how CUL4B regulates FOXO3A. FOXO3A had low expression when CUL4B scores were high, and high expression when CUL4B scores were low (A). (B) displays the negative correlation between CUL4B and FOXO3A in the total of SCC and SCLC tissues. (C) shows the negative correlation between CUL4B and FOXO3A in SCC. (D) displays the negative correlation between CUL4B and FOXO3A in SCLC. The mRNA level of FOXO3A was unchanged in CUL4B^knockdown^ cells (E). The activities of ERK and p-FOXO3A were downregulated in CUL4B^knockdown^ cells (F). PD98059 aggravated the decrease of p-ERK and p-FOXO3A in CUL4B^knockdown^ cells (G).

To evaluate the correlation between FOXO3A and CUL4B expression levels, we determined the expression of FOXO3A and CUL4B in SCC and SCLC tumor tissues by immunohistochemical staining. In **Figure 6A-D**, we found that the protein levels of CUL4B were inversely correlated with FOXO3A (*rs* = −0.6066, *P* < 0.001, *n* = 126) in SCC (*rs* = −0.6996, *P* < 0.001, *n* = 65) and in SCLC (*rs* = −0.5126, *P* < 0.001, *n* = 61) tissues. Upon further study, we detected the mRNA levels of FOXO3A in CUL4B^knockdown^ cells to examine whether FOXO3A was transcriptionally repressed by CUL4B. The results showed that there was no difference between sh-NT- and sh-CUL4B-treated cells (**Figure 6E**), indicating that that FOXO3A was not transcriptionally regulated by CUL4B, but p-FOXO3A (Ser 294) was decreased in CUL4B^knockdown^ cells as shown in **Figure 5A**. The decrease in H520 or H2227 was not as obvious as in SK-MES-1 and DMS-114; the reason might be that the phosphorylation of FOXO3A was not regulated by CUL4B. Therefore, the downregulation of p-FOXO3A (Ser 294) might be the reason why the depletion of CUL4B increased the protein level of FOXO3A.

ERK phosphorylates FOXO3A at Ser 294, and p-FOXO3A (Ser 294) increases the FOXO3A-MDM2 interaction and enhances FOXO3A degradation *via* an MDM2-dependent ubiquitin-proteasome pathway^24^. MDM2 expression is required in CUL4-mediated p53 polyubiquitination^29^. Hence, we determined whether CUL4B influenced the expression of FOXO3A from two aspects in SCC and SCLC cells. To test this hypothesis, we first determined if the activity of ERK was affected by CUL4B knockdown, if total ERK and p-ERK (an MAPK pathway target) were detected, and if PD98059 (an inhibitor of ERK kinases) could be used to inhibit ERK activity in CUL4B^knockdown^ cells. In **Figure 6F**, p-ERK was decreased, which was consistent with decreased p-FOXO3A in CUL4B^knockdown^ cells, but total ERK did not change. When ERK activity was inhibited, p-FOXO3A was further downregulated, and FOXO3A protein expression increased accordingly (**Figure 6G**).

**Figure 7:**
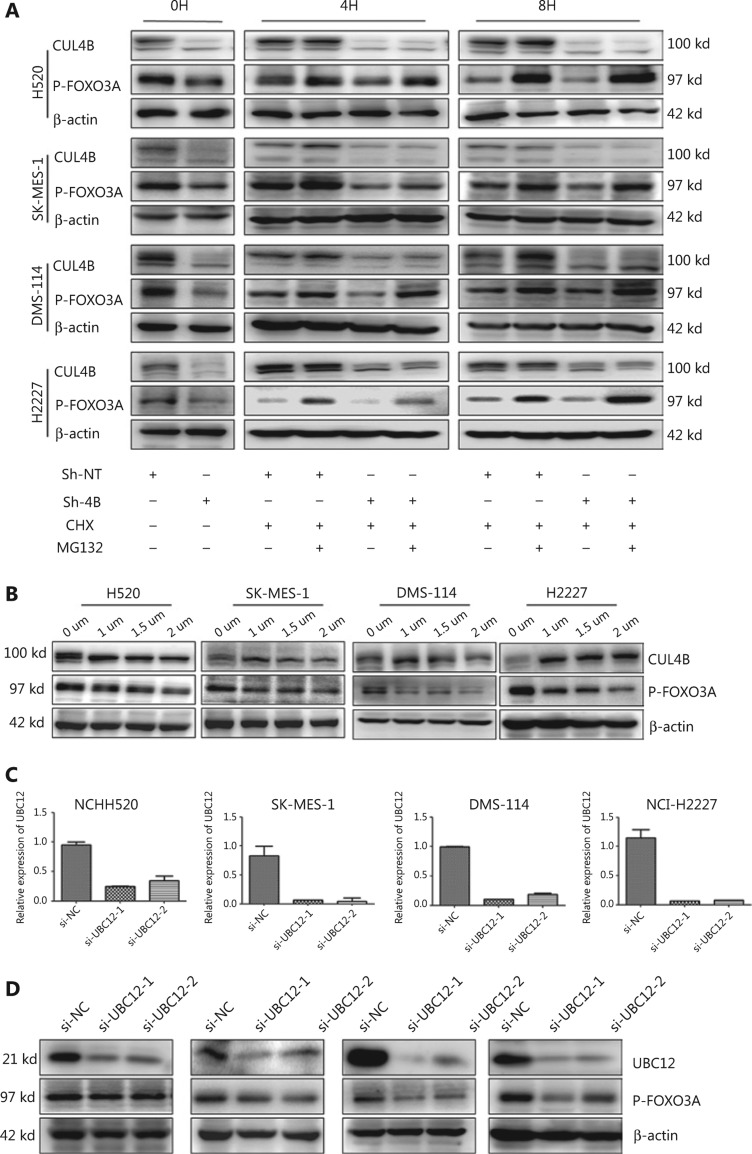
Depletion of CUL4B reduces the degradation of p-FOXO3A through the ubiquitin-proteasome system. MG-132 increased the protein level of p-FOXO3A (A). CUL4B activity inhibited by MLN4924 resulted in a decrease of p-FOXO3A (B). CUL4B activity inhibited by si-UBC12 resulted in a decrease of p-FOXO3A (C-D).

Second, to further validate FOXO3A degradation *via* the ubiquitin-proteasome pathway, MG-132 (a potent proteasome inhibitor) was used to suppress the activity of the proteasome in CUL4B^knockdown^ cells. The protein levels of p-FOXO3A were restored at either 4 or 8 h after MG-132 treatment (**Figure 7A**). Similar to other cullins, neddylation is essential for the activation of CUL4B^30^. Neddylation is a process in which the NEDD8 protein is conjugated to its target proteins, which relies on its own specific E1, E2, and E3 enzymes^31,32^. As further confirmation, MLN4924 (a NEDD8-activating enzyme inhibitor) was used to inhibit CUL4B activity. More importantly, the decreased levels of CUL4B activity were inversely correlated with the increased levels of p-FOXO3A (**Figure 7B**). These results suggested that the activation of CUL4B may be responsible for the decreased protein levels of FOXO3A. As expected, the cellular levels of p-FOXO3A were reduced in control siRNA-treated cells, while the neddylation pathway was blocked by the depletion of UBC12 (a NEDD8 E2-conjugating enzyme) (**Figure 7C and D**). These results indicated that the inactivation of CUL4B blocked the degradation of p-FOXO3A. Taken together, these results showed that CUL4B decreased the proapoptosis function of FOXO3A by activating the ERK signaling pathway and mediating p-FOXO3A degradation *via* the ubiquitin-proteasome pathway.

## Discussion

SCC is a common histological subtype of non-small cell lung cancer (NSCLC), and its development is associated with tobacco exposure, which is similar to SCLC^33,34^. Recently, rapid development of molecularly targeted drugs for patients with lung adenocarcinoma has greatly improved their prognoses. Unlike lung adenocarcinoma, therapy of SCC and SCLC still lacks safe, specific, and effective molecularly targeted drugs.

A growing number of studies have suggested that CUL4A and CUL4B are oncogenes. The aberrant expression of CUL4 in breast cancer and other malignant tumors compared to normal tissues supports its potential role in cancer development^17,35–37^. Several tumor suppressor genes, including p53, P21 and cyclin E, are ubiquitinated and degraded by CUL4A and CUL4B, which further supports the possibility of CUL4A and CUL4B as oncogenes^20,38,39^. Consistent with these reports, we showed that depletion of CUL4A and CUL4B inhibited SCC and SCLC cell proliferation. Notably, our study showed that CUL4A promoted cell proliferation by downregulating P21 to facilitate cell cycle processes, but CUL4B accelerated cell proliferation by downregulating FOXO3A to escape cell apoptosis in SCC and SCLC cells.

Studies of P21 in cigarette smoke exposure highlight its role in lung fibroblasts, which maintains its integrity of alveolar structure. Cigarette smoke might induce cell apoptosis or cell senescence of lung fibroblasts by promoting cell apoptosis *via* p53 or arresting cell proliferation *via* P21^40^. However, studies of P21 in cigarette smoke exposure-associated lung cancer have rarely been conducted. CUL4A is an inhibitor of nucleotide excision repair (NER), which repairs DNA damage^41^. Furthermore, CUL4A suppresses the G1-S DNA damage checkpoint by facilitating ubiquitin-dependent proteolysis of the rate-limiting NER factors, DDB2, XPC, and P21^42^. Our previous study consistently showed an inverse correlation between CUL4A and XPC or P21 in lung carcinoma patients^19^. Cytology experiments were then conducted, which showed that G1 phase arrest induced by CUL4A depletion was prevented in P21-CUL4A dual knockout cells.

**Figure 8:**
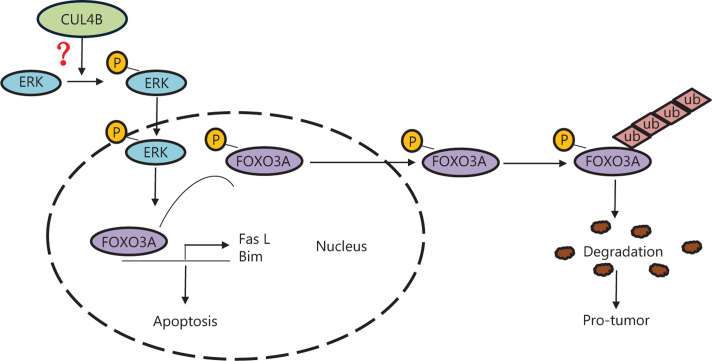
A working model of CUL4B-mediated FOXO3A degradation and cell apoptosis in lung cancer. The FOXO3a located in the nucleus promotes transcription of apoptosis-related genes such as *Bim*. CUL4B downregulates FOXO3 by promoting ERK phospho-activation (pERK) and subsequent phosphorylation of FOXO3A (p-FOXO3A), which is subject to degradation through the ubiquitin-proteasome pathway. However, the mechanism of CUL4B-mediated ERK activation still needs to be identified. It also remains possible that, as a key component of the E3 ubiquitin ligase machinery, the CUL4B complex may directly control FOXO3A stability.

CUL4B, as an E3 ligase, has two different functions: traditional ubiquitin-proteasome-dependent protein degradation and nuclear shuttling to exert transcriptional repression of genes. CUL4B is negatively correlated with the level of IGFBP3 expression, which is induced by wild-type p53, and which enhances the p53-dependent apoptotic response to DNA damage^18,43,44^. However, in the present study, because FOXO3A promoted apoptosis and was transcriptionally suppressed by CUL4B, we detected FOXO3A-mediated apoptosis in SCC and SCLC cells. First, we confirmed that FOXO3A was a crucial factor in CUL4B knockdown-induced cell apoptosis. The mechanism of FOXO3A regulation by CUL4B from two aspects was then clarified. CUL4B decreased FOXO3A expression by activating the ERK signaling pathway and mediating p-FOXO3A degradation *via* the ubiquitin-proteasome pathway. The present study is therefore the first to report that CUL4B is an anti-apoptosis molecule, which occurs partially through activation of the ERK signaling pathway and phosphorylation of FOXO3A in lung cancer (the whole pathway is summarized in **Figure 8**). However, further experimentation is needed to define the detailed mechanism of how CUL4B influences ERK signaling pathway.

## Conclusions

We found that depletion of CUL4A resulted in the accumulation of P21 and thereby inhibited the G1/S transition. However, the depletion of CUL4B resulted in an impaired pERK-mediated FOXO3A phosphorylation and degradation, which eventually promoted cell apoptosis. Our findings provide evidence for future cancer therapy by interference with CUL4A or CUL4B. Furthermore, we identified the specific roles of CUL4A and CUL4B in lung cancer.
